# Intraspecific variation in oxidative stress tolerance in a model cnidarian: Differences in peroxide sensitivity between and within populations of *Nematostella vectensis*

**DOI:** 10.1371/journal.pone.0188265

**Published:** 2018-01-26

**Authors:** Lauren E. Friedman, Thomas D. Gilmore, John R. Finnerty

**Affiliations:** Department of Biology, Boston University, Boston, MA, United States of America; University of California Irvine, UNITED STATES

## Abstract

*Nematostella vectensis* is a member of the phylum Cnidaria, a lineage that includes anemones, corals, hydras, and jellyfishes. This estuarine anemone is an excellent model system for investigating the evolution of stress tolerance because it is easy to collect in its natural habitat and to culture in the laboratory, and it has a sequenced genome. Additionally, there is evidence of local adaptation to environmental stress in different *N*. *vectensis* populations, and abundant protein-coding polymorphisms have been identified, including polymorphisms in proteins that are implicated in stress responses. *N*. *vectensis* can tolerate a wide range of environmental parameters, and has recently been shown to have substantial intraspecific variation in temperature preference. We investigated whether different clonal lines of anemones also exhibit differential tolerance to oxidative stress. *N*. *vectensis* populations are continually exposed to reactive oxygen species (ROS) generated during cellular metabolism and by other environmental factors. Fifteen clonal lines of *N*. *vectensis* collected from four different estuaries were exposed to hydrogen peroxide. Pronounced differences in survival and regeneration were apparent between clonal lines collected from Meadowlands, NJ, Baruch, SC, and Kingsport, NS, as well as among 12 clonal lines collected from a single Cape Cod marsh. To our knowledge, this is the first example of intraspecific variability in oxidative stress resistance in cnidarians or in any marine animal. As oxidative stress often accompanies heat stress in marine organisms, resistance to oxidative stress could strongly influence survival in warming oceans. For example, while elevated temperatures trigger bleaching in corals, oxidative stress is thought to be the proximal trigger of bleaching at the cellular level.

## Introduction

The starlet sea anemone, *Nematostella vectensis*, is a leading laboratory model organism in the phylum Cnidaria due to its ease of culture, amenability to a wide range of experimental protocols, and the availability of abundant genomic resources [1–19]. In its natural habitat—estuaries along the Atlantic coast of North America—this animal experiences dramatic variation in key environmental variables over short temporal and spatial scales [20]. Furthermore, there is extensive genetic and phenotypic variation between populations of *N*. *vectensis* [21–27]. Atlantic coast populations have a high degree of genetic differences and regional population genetic structure that is not correlated to relative geographic distances [21, 25]. These differences within local populations are hypothesized to be the result of limited gene flow within and between *N*. *vectensis* populations, adaptation to local environmental conditions, and episodic anthropogenic dispersal over large distances. The estuarine habitats inhabited by *N*. *vectensis* are often physically isolated, and these habitats can differ dramatically with respect to key environmental parameters, such as temperature, pH, and salinity [3, 20]. Moreover, the early stages of the *N*. *vectensis* lifecycle exhibit limited dispersal ability, due in part to the egg mass being negatively buoyant [3]. The combination of diverse environments and limited dispersal will tend to promote adaptive genetic differentiation between populations. For this reason, *N*. *vectensis* has been advocated as a useful model for studying adaptive microevolution in a coastal invertebrate beset by rapid environmental change [6, 24].

One environmental stressor that varies dramatically in salt marshes and other estuarine habitats is hydrogen peroxide (H_2_O_2_) concentration. For example, in an intertidal salt-flat off the coast of Germany, H_2_O_2_ concentrations in the water were found to vary almost 100-fold, from 0.05 μM to approximately 4.5 μM, with concentrations at the sediment-water boundary layer being 2–4 times higher than concentrations in the adjacent bottom water [28]. Peroxide levels can accumulate in shallow coastal habitats due to natural inputs such as photochemical production and atmospheric wet deposition as well as anthropogenic inputs such as runoff from industrial processes, e.g., mining and industrial manufacturing. Salt marshes are particularly susceptible to peroxide accumulation because large amounts of organic matter can fuel peroxide production through photo-degradation [29], and isolated pools may seldom be flushed.

*N*. *vectensis* is a benthic salt-marsh organism with relatively low mobility, so this species is readily exposed to compounds that accumulate in sediments of estuaries. In its natural habitat, *N*. *vectensis* can be exposed to a wide range of exogenously produced reactive oxygen species (ROS) including H_2_O_2_. This anemone must, therefore, be under strong selection to counteract oxidative stress. *N*. *vectensis* is known to tolerate a wide range of other highly variable environmental parameters, e.g., salinities from ~0–50 ppt and temperatures from 2–28.5°C [3, 20]. The ability to tolerate rapid and dramatic variations in these environmental conditions suggests that individual *N*. *vectensis* have wide environmental tolerances. At the same time, the pronounced environmental differences between and within estuaries inhabited by *N*. *vectensis* suggest the potential for local adaptation to differing selection pressures. The potential for local adaptation is increased by the fact that *N*. *vectensis*’ natural dispersal ability appears limited, as evidenced by significant genetic structure between and within estuaries [21, 25]. Specifically with reference to peroxide tolerance, one might expect that all *N*. *vectensis* would exhibit a wide tolerance to peroxide, with no observable differences between or within populations. However, given that a recent study identified substantial differences in temperature-specific growth and regeneration rates in *N*. *vectensis* [24], we sought to determine whether different clonally derived lines of anemones would also exhibit differential peroxide tolerance. Indeed, as elevated temperatures promote oxidative stress (e.g., [30]), we hypothesized that the same clonal lines that are best adapted to high temperatures would also be most resistant to oxidative stress.

In this study, we exposed fifteen clonal lines of *N*. *vectensis* collected from four geographically isolated estuaries to different levels of H_2_O_2_. Our study included three clonal lines (one each from Baruch, South Carolina, Meadowlands, New Jersey, and Kingsport, Nova Scotia) known to vary in their growth and/or regeneration rates at 21 and 29°C [24], as well as 12 previously uncharacterized clonal lines established from founder individuals collected in Great Sippewissett Marsh, Falmouth, MA. We monitored the effects of H_2_O_2_ on adult survival and on the ability of animals to regenerate a new “head” following bisection through the body column. We identified substantial differences in the effects of peroxide on survival and regeneration between the SC, NJ, and NS clonal lines and among clonal lines originating from the single estuary in Sippewissett.

## Materials and methods

### Animal collection and husbandry

The clone lines used in this study were derived from individual field-caught anemones collected prior to 2008 for a population genetic study from estuaries in four locations: Kingsport, Nova Scotia; Great Sippewissett Marsh, Falmouth, MA; Meadowlands, NJ; and Baruch, SC [25]. Within Sippewissett Marsh, founder individuals were collected from three distinct pools designated Pool A, B and C. Shortly after capture, individual anemones were isolated to separate culture containers and then used to establish clonal lines by repeatedly bisecting the animals and allowing them to regenerate. All clonal lines were housed in the laboratory using standard culture conditions [1] for at least five years before being used in this study. The clone lines used in this study were previously tested for their temperature-specific growth and regeneration rates [24].

### Preliminary peroxide exposure tests

The levels of peroxide used in our experiments were determined empirically. In a pilot experiment conducted by J. C. Sullivan in the Finnerty lab [31], multiple strains of *N*. *vectensis*, including the Nova Scotia, New Jersey and South Carolina clone lines tested in this study, were challenged to regenerate under a range of H_2_O_2_ concentrations from 0.0001–0.01% (32.6–3260 μM). At concentrations exceeding 0.0005% (163 μM) H_2_O_2_, anemone mortality reached 100%. At 0.0005% H_2_O_2_, some anemones survived the exposure, albeit while exhibiting a pronounced delay in the regeneration rate relative to control conditions. Thus, for the current study, we characterized adult survivorship at 0.0005% H_2_O_2_, while we evaluated regeneration rate at half that concentration (0.00025% or 82 μM)_._

### Peroxide survival experiments

Several individual anemones from each clone line (n = 5–10) were maintained in 0.0005% (163 μM) H_2_O_2_/11 ppt artificial seawater (ASW; Instant Ocean™) or under control conditions (11 ppt artificial seawater) for a period of two weeks. The ASW/H_2_O_2_ was made fresh daily, and both ASW/H_2_O_2_ and ASW media were changed daily. Over the course of these experiments, each individual anemone was housed in a single well of a 12-well tissue-culture plate (Thermo Scientific Nunc) and maintained at 20°C on a 12 h-light/12 h-dark diel cycle. Once per day, individual anemones were scored for tentacle number and for the ability to contract the tentacles and body column in response to mechanical stimulation. Single factor ANOVAs were performed to identify significant differences in average tentacle number between peroxide-exposed and control individuals on each day of the study (p<0.05). Animals that had lost the ability to respond to mechanical stimulation were scored as deceased. All surviving anemones that had been subjected to peroxide exposure were retired from future stress-tolerance studies due to concerns over potential lingering epigenetic effects of exposure.

### Peroxide regeneration experiments

Individual anemones from each clonal line (n = 10–20) were bisected along the transverse axis at the midpoint of the body column, approximately equidistant from the tip of the foot and the opening of the mouth [1]. The aboral halves of 50% of the bisected anemones (n = 5–10) from each clonal line were then transferred to 0.00025% (82 μM) H_2_O_2_/11ppt ASW (i.e., half of the H_2_O_2_ concentration used in the survival experiments). These animals were then allowed to regenerate for 14 days, while the other 50% were allowed to regenerate for the same period under control conditions (11 ppt ASW). The H_2_O_2_/ASW or ASW media were changed daily. The anemones were sorted between H_2_O_2_ and control treatments so that mean body length was approximately equal between treatments. Individuals used in these experiments measured 0.5–3.0 cm in length from the tip of the foot (or physa) to the oral opening. Regenerating anemones were scored daily for the presence and number of tentacles, and after 14 days, they were scored for their ability to feed. Previous studies have shown that the regeneration process is slowed when the bisection is not clean or the mesenteries are exposed [32]; therefore, if the bisection site of the regenerating anemones was not a clean cut, then the individual was not included in the study. ANOVAs were performed to determine significant differences in average tentacle number during regeneration between H_2_O_2_-exposed and control individuals. As with the H_2_O_2_ survival experiments, all surviving anemones that successfully regenerated when exposed to H_2_O_2_ were retired from any future stress-tolerance studies due to concerns over potential lingering epigenetic effects of exposure.

## Results

### Differences between genets in adult survivorship upon exposure to H_2_O_2_

**Fig 1:**
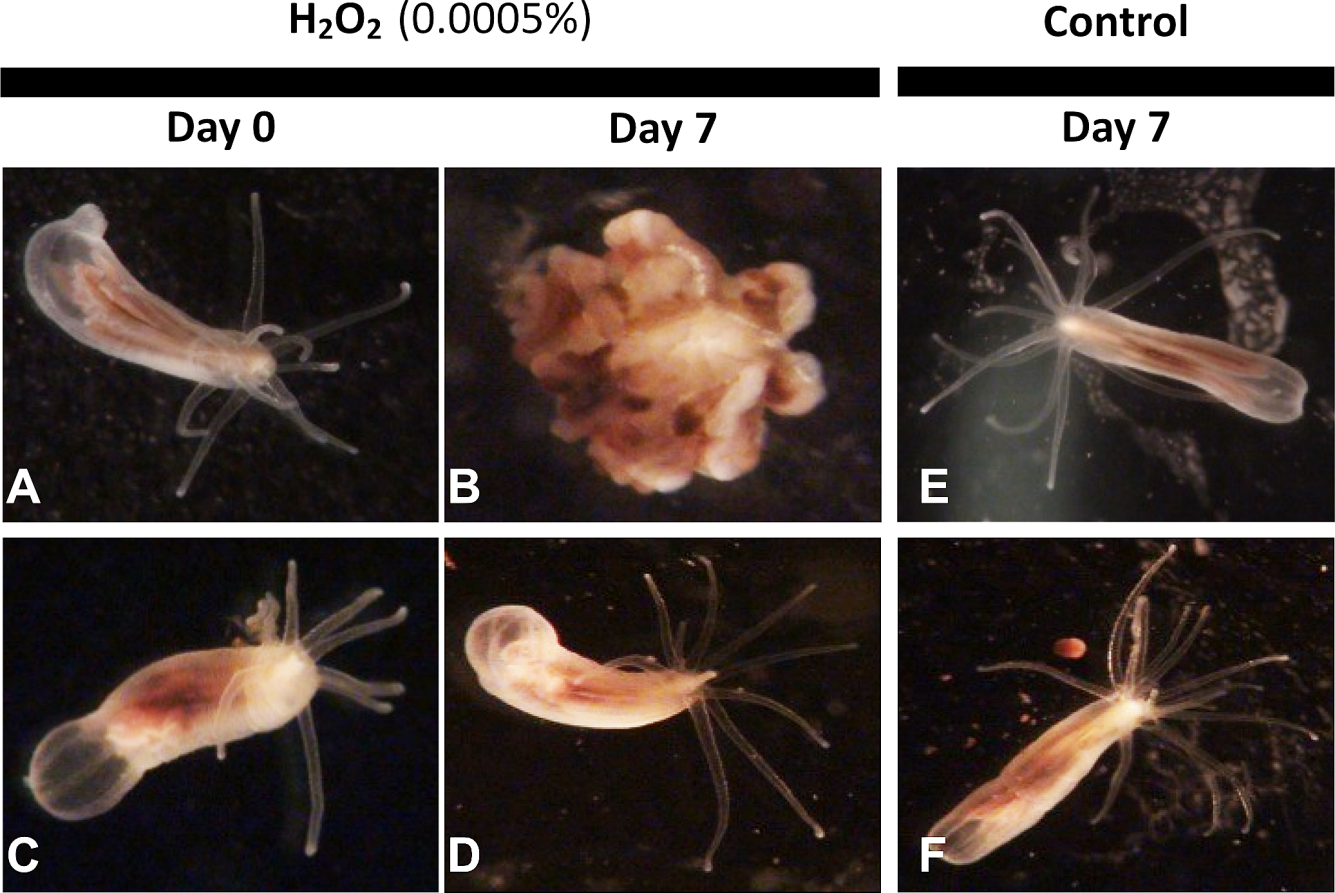
Representative adult tolerance to H_2_O_2_ exposure. Representative individuals from Sippewissett MA (top row: clone line MA B1; bottom row: clone line MA A1) during exposure to 0.0005% H_2_O_2_ (A-D) or control conditions (E-F) over 7 days. After a week, the majority of the clonal lines showed severe disfigurement and loss of tentacles (as in B), but some clonal lines appeared relatively resistant to H_2_O_2_ exposure (as in D).

**Fig 2:**
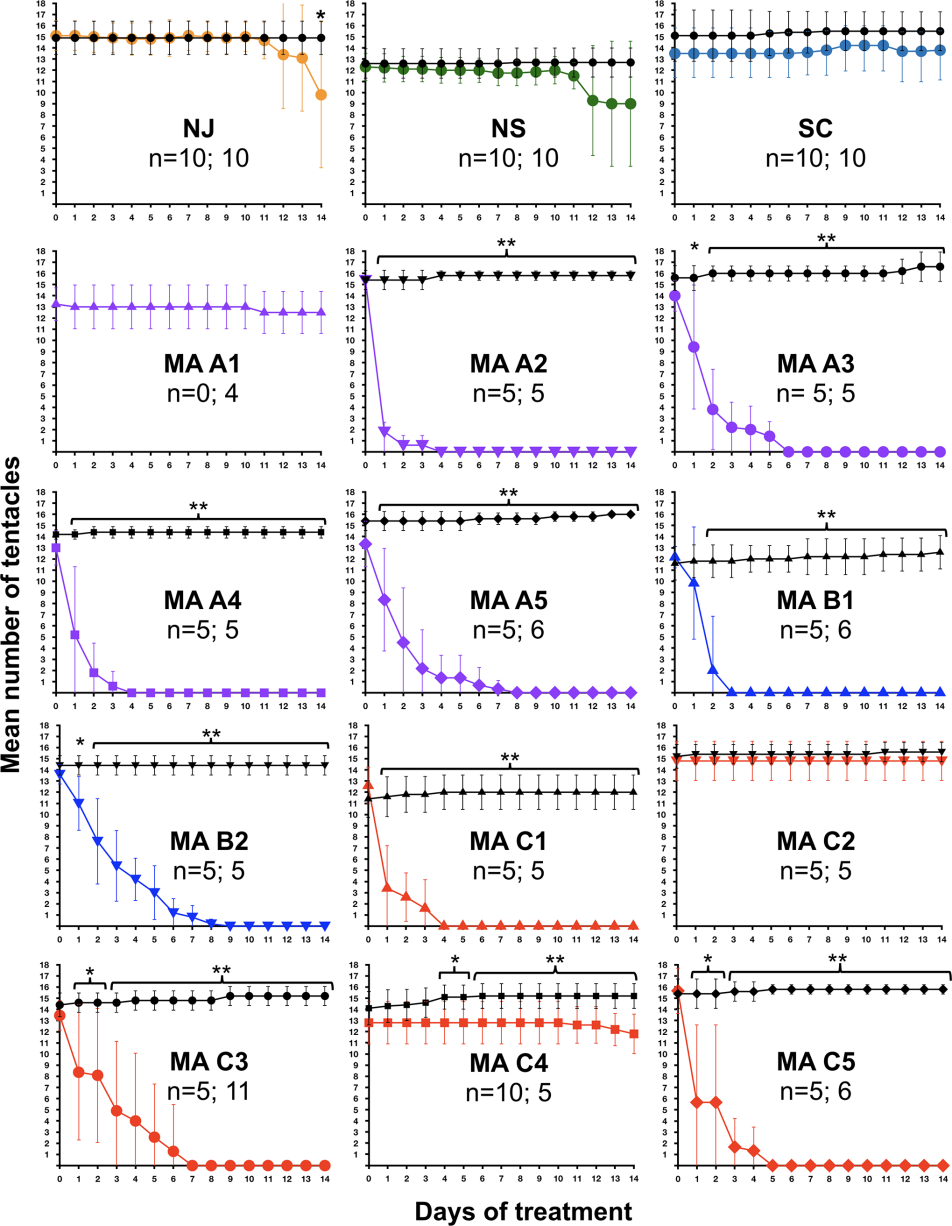
Tentacle number during 14-day exposure to H_2_O_2_ or control conditions. Data for 15 *N*. *vectensis* clone lines are shown. In all graphs, days of exposure are given along the X-axis, and tentacle numbers are shown on the Y-axis. Data from the control condition are shown in black, while data from H_2_O_2_ exposure are shown in colored lines. The number of individuals tested under control conditions and H_2_O_2_ exposure is provided for each clonal line (n = #/#). Statistically significant differences between H_2_O_2_ and control conditions on a given day are indicated using asterisks (* = p<0.05; ** = p<0.005). Error bars depict the standard deviations for each mean.

**Fig 3:**
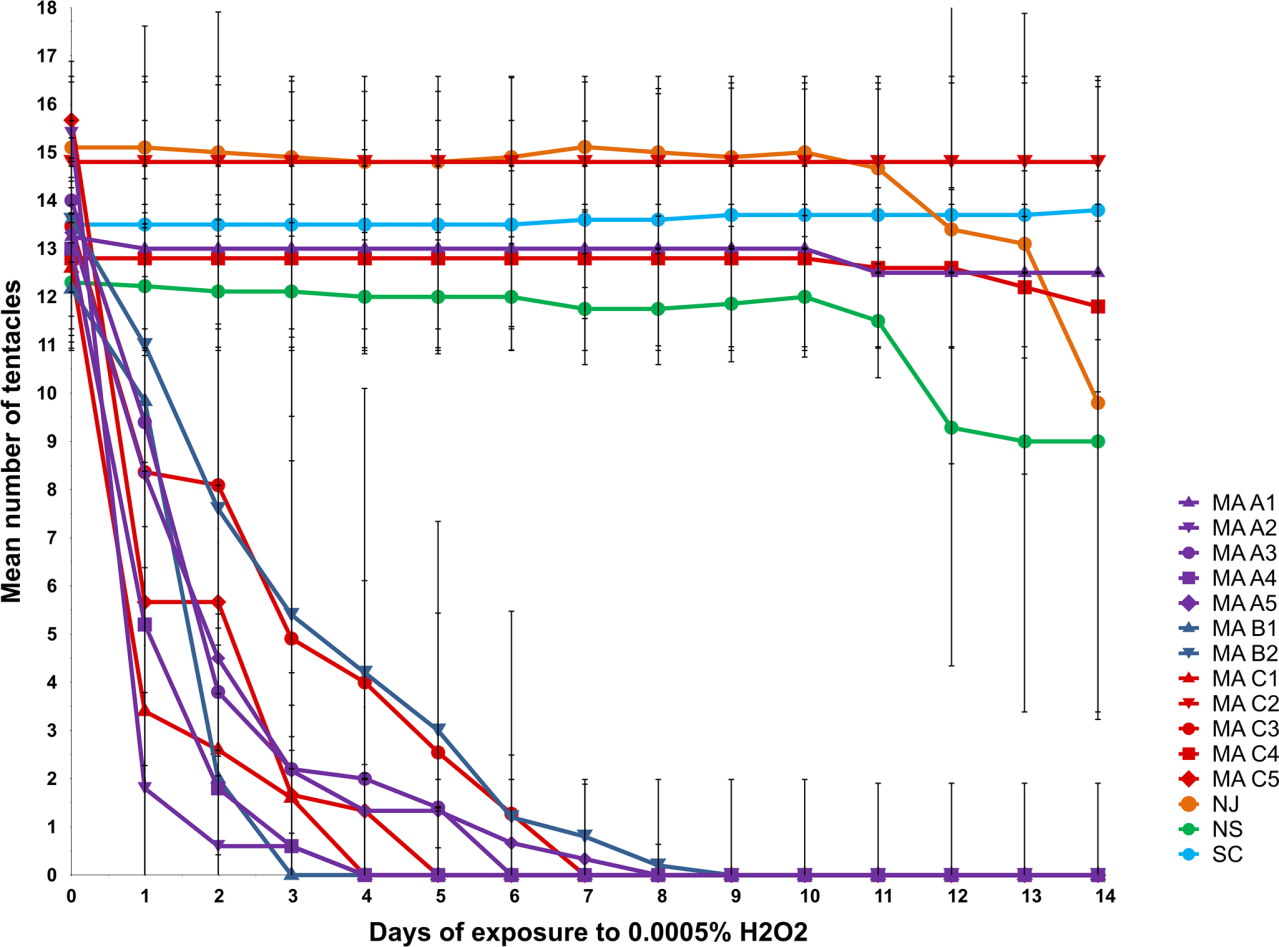
Mean tentacle counts during H_2_O_2_ exposure. For each of 15 *N*. *vectensis* clonal lines, mean tentacle number for 4–11 individuals is plotted against days of exposure to 0.0005% H_2_O_2_. Thin vertical lines depict standard errors.

To determine whether anemones varied in their tolerance to H_2_O_2_, over a period of 14 days, we compared the number of tentacles in adult polyps incubated in various concentrations of H_2_O_2_ to those in anemones in control conditions (Fig 1). We used this metric as an indicator of the anemones’ condition because tentacle number is easy to score, and the tentacles are particularly sensitive to tissue damage from elevated H_2_O_2_. Furthermore, it is necessary for the tentacles to be extended during feeding, and tentacles are typically extended in unstressed animals. In all clone lines tested, there were no changes in mean tentacle numbers in control conditions. However, relative to controls, there was a significant decrease in tentacle number in several clonal lines under conditions of H_2_O_2_ exposure (Figs 2 and 3). In the clonal line from Nova Scotia (NS), mean tentacle number remained generally steady over the first ten days of peroxide exposure, but declined on days 11–12, and remained lower than in control anemones, though the difference was not statistically significant. In the clonal line from New Jersey (NJ), H_2_O_2_-exposed and control animals began to diverge in tentacle number at day 12, and the difference between treatments became statistically significant by day 14. In the South Carolina clonal line, neither a decline in tentacle number nor any visible tissue disfigurement was observed over the course of the treatment period.

Differences between the NJ, NS, and SC clonal lines in their response to elevated H_2_O_2_ were not surprising given previously demonstrated differences in their response to elevated temperatures [24]. However, we observed more substantial differences in H_2_O_2_ tolerance among 12 *N*. *vectensis* clonal lines generated from individuals collected from three separate pools in Sippewissett Marsh, MA in 2008 (Figs 2 and 3). In particular, three Sippewissett clonal lines appeared minimally affected by H_2_O_2_ exposure (MA-A1, MA-C2, and MA-C4), while the other nine clonal lines displayed varying levels of disfigurement and loss of tentacles over time (Figs 2 and 3). The three clonal lines minimally affected by H_2_O_2_ exposure had been collected from two different pools in Sippewissett Marsh. Two of the resistant clonal lines came from Pool C, which also contained three clonal lines that were sensitive to H_2_O_2_ exposure. The other resistant clonal line came from Pool A (MA-A1), and there were four sensitive clonal lines also from that pool (MA-A2, A3, A4 and A5). Unfortunately, we are unable to directly compare the performance of MA-A1 under H_2_O_2_-exposed versus control conditions because this clonal line perished, apparently due to disease, before the control could be performed.

### Regeneration is differentially affected by H_2_O_2_ in different genets

**Fig 4:**
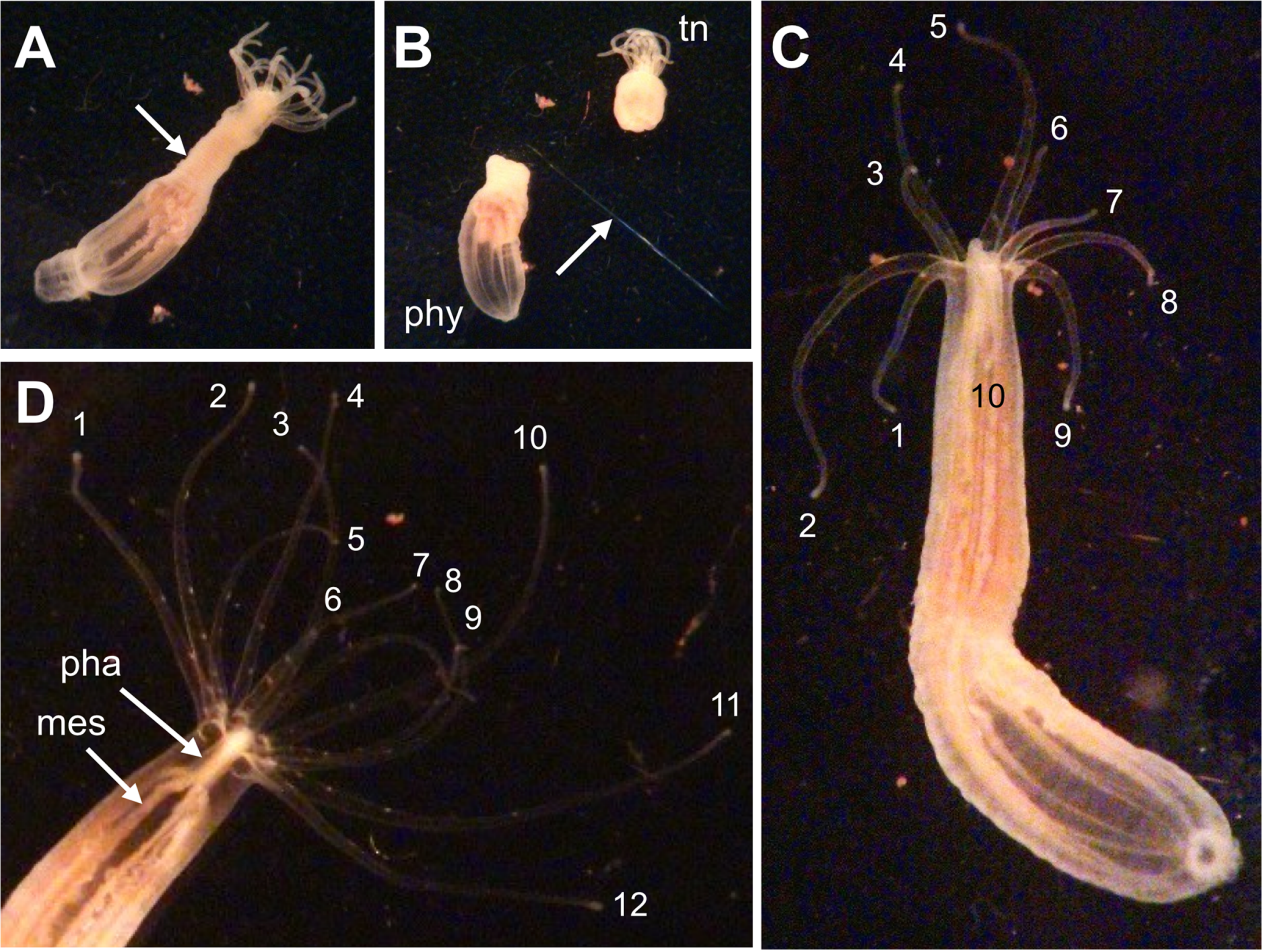
Representative regenerating *N*. *vectensis* during H_2_O_2_ exposure. An adult *N*. *vectensis* from New Jersey, approximately 1 cm in length (A), was bisected through the body column (at the site of the arrow) using a scalpel. The cut divided the anemone into (B) an oral fragment bearing the mouth and tentacles (tn) and an aboral fragment bearing the foot, or physa (phy). The scalpel scored the bottom of the plastic petri dish in which the bisection was performed (arrow). Following bisection, the regenerating aboral fragment was exposed to 0.00025% H_2_O_2_ over a period of 14 days. Seven days after bisection (C), the aboral fragment had developed 10 tentacles. The tentacle tips are numbered in the photo. By day 14 (D), 12 tentacles had developed. In panel D, the pharynx (pha) and several of the eight complete mesenteries (mes) are visible.

**Fig 5:**
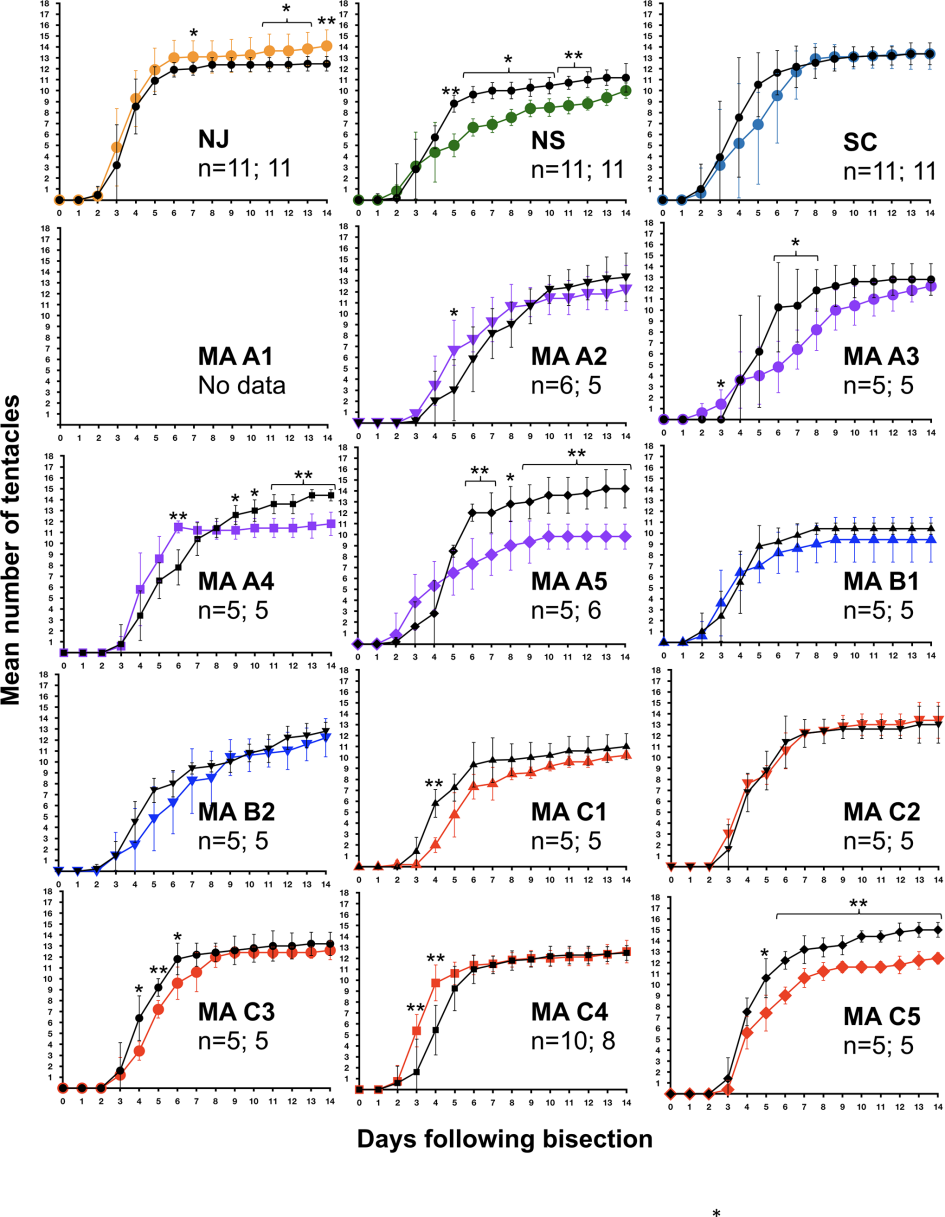
Tentacle counts in regenerating *N*. *vectensis*. Regeneration ability as measured by mean tentacle number over time (n = 5–11) during exposure to 0.00025% H_2_O_2_ or control conditions. In all graphs, the number of days since the anemones were bisected is indicated along the X-axis, and tentacle number is shown on the Y-axis. Data from the control condition are shown in black, while data from H_2_O_2_ exposure are shown in colored lines. The number of individuals tested under control conditions and H_2_O_2_ exposure is provided for each clonal line (n = #/#). Statistically significant differences between H_2_O_2_ and control conditions on a given day are indicated using asterisks (* = p<0.05; ** = p<0.005).

To determine whether H_2_O_2_ had an effect on regeneration, we bisected animals, isolated the aboral halves, and then counted the number of tentacles in these regenerating animals once per day over a 14-day period under control conditions or in the presence of H_2_O_2_ (Fig 4). In some clonal lines, the rate of regeneration was significantly affected by H_2_O_2_ exposure (Fig 5). The NS clonal line showed a significant effect of H_2_O_2_ exposure on average number of tentacles present on days 5–12 (p<0.05), with highly significant effects on days 5, 11, and 12 (p<0.005). The H_2_O_2_-exposed NJ individuals showed significantly lower average tentacle numbers on day 7 as well as days 11–14 of regeneration (p<0.05), with highly significant differences apparent by day 14 (p<0.005). The SC clone line showed no statistically significant effects of H_2_O_2_ exposure on tentacle number throughout the 14-day regeneration period.

Continual H_2_O_2_ exposure had varying effects on regeneration in 11 clonal lines from Sippewissett Marsh (Fig 5). In general, the ability to regenerate in 0.00025% H_2_O_2_ was correlated with the ability to survive in 0.0005% H_2_O_2_. From MA pool A, the four clonal lines that were sensitive to H_2_O_2_ in the adult survival assay (MA-A2, A3, A4, A5) also showed delayed regeneration in the presence of H_2_O_2_ at some time points. In clonal lines MA-A4 and A5, a significant detrimental effect of H_2_O_2_ was observed until the end of the study, becoming increasingly severe over the 14-day period. In MA-A2 and A3, the tentacle counts of individual anemones undergoing H_2_O_2_ treatment were similar to the mean tentacle numbers of the control specimens by the end of the study. Unfortunately, the clonal line from pool A that exhibited the greatest tolerance to H_2_O_2_ in the adult survival assay (MA-A1) was lost due to an apparent disease before it could be subjected to the H_2_O_2_ /regeneration assay. While neither of the two clonal lines from pool B was able to tolerate 0.0005% H_2_O_2_ as an adult, both were able to regenerate without any detectable effect in 0.00025% H_2_O_2_. From pool C, in the two clonal lines that demonstrated the greatest H_2_O_2_ tolerance as adults (MA-C4 and C2), there were also negligible or minor effects of H_2_O_2_ on regeneration. In MA-C2, regeneration was not affected by H_2_O_2_, whereas in MA-C4, regeneration was affected by H_2_O_2_ on days 3 and 4, but H_2_O_2_-treated individuals were indistinguishable from control anemones on days 5–14. Some of the less resistant clones (MA-C1 and C3) showed delayed rates of tentacle development early in regeneration (e.g., day 4), but were similar to control animals by the end of the study. By contrast, clonal line MA C5 was the most sensitive to H_2_O_2_, as it showed the most persistent and severe detrimental effects of H_2_O_2_ on regeneration, with a highly significant (p<0.005) decrease in average tentacle number versus control animals from days 8–14.

**Fig 6:**
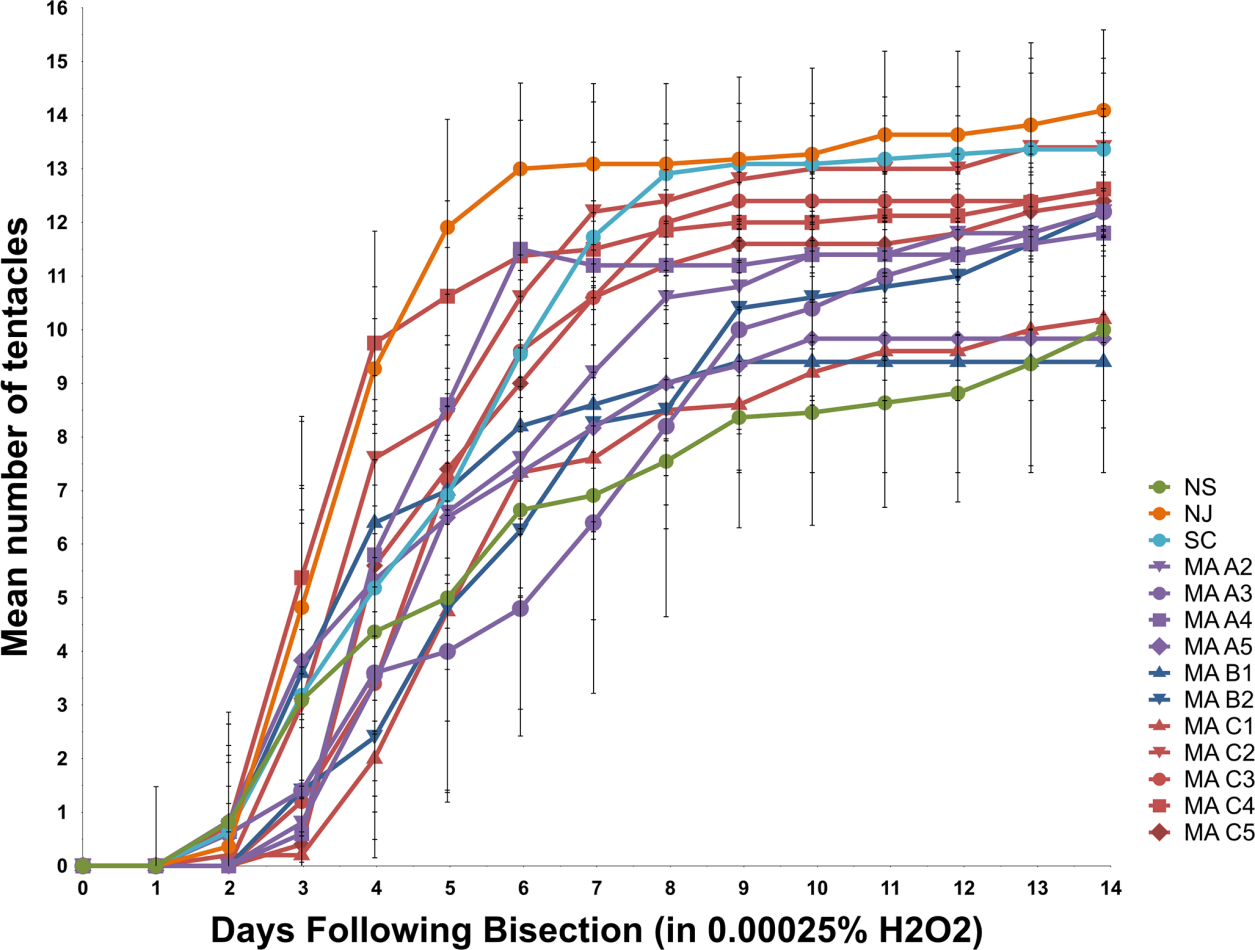
Mean tentacle counts during regeneration under H_2_O_2_ exposure. For each of 14 *N*. *vectensis* clonal lines, mean tentacle number for 5–11 individuals (see Fig 6) is plotted against days of exposure to 0.00025% H_2_O_2_. Thin vertical lines depict standard deviations.

In a comparison of the performance of all clonal lines in the presence of H_2_O_2_ (Fig 6), the greatest variation in tentacle number was observed on days 4–6. For example, on day 4, the clonal line with the lowest mean tentacle count (MA-C1) averaged fewer than two tentacles per individual anemone, whereas the clonal line with the highest mean tentacle count (MA-C4) averaged approximately ten tentacles per individual. By day 14, many clonal lines appeared to achieve a steady-state number of tentacles ranging between ~9 (MA-B1) and ~14 (NJ).

## Discussion

In this study, we applied exogenous H_2_O_2_ to assess the oxidative stress response of the estuarine sea anemone *Nematostella vectensis*. This is a straightforward and biologically relevant way to challenge the oxidative stress response of a coastal marine invertebrate because organisms in shallow coastal waters regularly encounter elevated H_2_O_2_ levels that can trigger oxidative stress (e.g., [28]). Furthermore, peroxide is the most abundant and long-lived ROS in marine environments, and unlike other ROS, H_2_O_2_ is uncharged and can diffuse freely across membranes (reviewed in [33]).

The peroxide levels used in our experiments (82 and 163 μM) are substantially higher than those recorded in marine and freshwater environments (Table 1). Across numerous ocean basins, the peroxide concentration of sea surface waters have been found to range from 10–420 nM [34–36], while peroxide concentrations in the surface waters of lakes have been found to exceed 1 μM (*e*.*g*., [37]). At a site more comparable to the habitat of *N*. *vectensis*, peroxide levels in a shallow tidal pool located on a sand flat, ~500 m off the coast of Germany were found to vary between 0.5 and 4.5 μM [28]. However, in contrast to the high marsh habitat in which the anemones used in the current study were collected, this sand flat is inundated by the tides for ~19 h each day [28]. Of note, the levels of H_2_O_2_ in rainwater can be substantially higher than the surface waters of oceans or lakes, with continental rainwater ranging from 0.1–247 μM (reviewed in [38, 39]) and oceanic rainwater ranging from 3.5 to 82 μM [40, 41]. As a result, precipitation events can substantially increase H_2_O_2_ levels in surface waters [40–42].

**Table 1:** Hydrogen peroxide levels measured in aquatic environments.

Geographic locale	Descriptions	Max [H_2_O_2_]	Ref.
Gulf of Mexico off the western coast of Florida	Sea surface waters to 200 m	~0.024 x 10^−6^ M	[43]
Wadden coast, Germany	Shallow tidal pool ~500 m from land	~4.5 x 10^−6^ M	[28]
King George Isl., Antarctica	Offshore surface waters	~1.8 x 10^−6^ M	[68]
	Intertidal pools	~2.0 x 10^−6^ M	
	Freshly fallen snow	~13.6 x 10^−6^ M	
Jacks Lake, Ontario, CA	Oligotrophic near-shore lake water	~0.8 x 10^−6^ M	[69]
	Continental rain	34 x 10^−6^ M	
Gulf of Mexico	Marine rain	82 x 10^−6^ M	[40]

While H_2_O_2_ levels have not yet been reported for any saltmarsh, we suspect that the pools inhabited by *N*. *vectensis* regularly experience higher concentrations than those typically found in oceans or lakes. The saltmarsh pools where the anemones in this study were collected are small, shallow bodies of water. As such, they receive proportionately large inputs of H_2_O_2_ through precipitation. Snow and rain can both have much higher peroxide concentrations than seawater (Table 1), and in shallow, stratified layers of marine surface waters, rain events have been shown to increase the H_2_O_2_ concentration by more than 10-fold [40]. Furthermore, saltmarsh pools receive abundant direct sunlight and are rich in organic matter. As a result, the production of H_2_O_2_ via the photochemical decay of organic matter, which is thought to be the principal source of H_2_O_2_ in surface waters [29, 43], should be particularly high. Furthermore, saltmarsh pools, particularly those at higher elevation, may only rarely be inundated by the tides, so their H_2_O_2_ content would not often be diluted by an influx of seawater. Finally, in the upper layer of the sediment, where the anemones burrow, photosynthetic algae and bacteria can generate locally high levels of oxygen [44] and concomitantly high levels of ROS.

The effects of H_2_O_2_ exposure have been studied in a handful of marine invertebrates, including polychaete worms, molluscs, crustaceans, and corals, but the concentrations of peroxide that were used and the effects that were monitored have varied substantially among these studies (Table 2). In whole-organism studies, micromolar concentrations of H_2_O_2_ (0.5–20 μM) were shown to decrease oxygen consumption and aerobic metabolism in three intertidal species, a burrowing polychaete [45], a clam [46], and a limpet [47]. Five micromolar H_2_O_2_ also caused a substantial drop in water filtration rate in an intertidal clam [46]. A higher level of H_2_O_2_ (50 μM) was used in experiments on the estuarine polychaete, *Laeonereis acuta*. In this infaunal worm, peroxide exposure caused lipid peroxidation and DNA damage [48]; there was also a biphasic effect on aerobic metabolism, with O_2_ consumption falling after 4 d exposure and rising again after 7 d [49]. In the tropical reef coral, *Galaxea fascicularis*, exposure to 0.3 or 3 μM H_2_O_2_ was accompanied by an increase in catalase activity, but no increase in superoxide dismutase activity [50], and this pattern was observed at both 27°C and 31°C [51]. In comparison to our study, all of these organismal investigations utilized lower levels of H_2_O_2_, and they did not examine tissue integrity, mortality, or a developmental process (such as regeneration). Significantly higher levels of peroxide have been applied to an isolated body-wall preparation obtained from an intertidal polychaete (42–664 μM; [52]) and to a mitochondrial suspension from a sipunculid (90.9 μM; [53]). Finally, millimolar levels of H_2_O_2_ have been shown to induce synchronous spawning in molluscs in a study that did not investigate subsequent impacts of H_2_O_2_ exposure on survivorship or physiological function [54]. In studies on cultured cells, H_2_O_2_ levels comparable to or greater than those employed here have been used to induce “mild” oxidative stress; for example, Huang et al. exposed HeLa cells to 1000 μM H_2_O_2_ for 1 h or 100 μM H_2_O_2_ for 6 hours to investigate its effects on protein localization [55]. Of note, our study demonstrates the highest level of survivable peroxide exposure that has been found to date for any marine or estuarine invertebrate.

**Table 2:** Effects of hydrogen peroxide exposure on marine invertebrates.

Organism	Habitat / Organismal description	Peroxide Level Tested	Effect(s) of exposure	Ref.
*Galaxea fascicularis*	Tropical reef coral (Cnidaria/Anthozoa)	0.3; 3 μM	Increased catalase activity	[50]
		0.3; 3 μM + 27/31°C	Increased catalase activity in coral host and symbiotic zooxanthellae at either temp.	[51]
*Nereis diversicolor*	Intertidal burrowing worm (Polychaeta)	0.5; 5 μM	Locomotion ceases; O_2_ consumption was reduced by ~40%	[45]
*Cerastoderma edule*	Intertidal clam (Bivalva)	5 μM	Filtration rates reduced by 40%	[46]
*Nacella concinna*	Antarctic intertidal limpet (Gastropoda)	5 μM	Lysosomal membrane instability; 45% reduction in aerobic metabolism	[47]
*Crangon crangon*	Intertidal shrimp (Crustacea)	20 μM	O_2_ uptake reduced by 25.7%; decrease in muscle intracellular pH	[70]
*Laeonereis acuta*	Infaunal estuarine worm (Polychaeta)	10; 50 μM	Lipid peroxidation; DNA damage;	[49]
			Initial fall (at 4 d) and later rise (at 10 d) in O_2_ consumption	[48]
*Sipunculus nudus*	Intertidal burrowing worm (Sipuncula) mitochondrial suspension.	90.9 μM	Did not inhibit ATP synthesis	[53]
*Arenicola marina*	Intertidal burrowing worm (Polychaeta); isolated body-wall prep	42 μM 328 μM 664 μM	17% increase in O_2_ consumption 17% decrease in O_2_ consumption 9% decrease in O_2_ consumption	[52]

While previous studies on oxidative stress tolerance in marine animals have generally sought to characterize a species’ average or typical response to a given challenge, our study is the first to examine intraspecific variation in the oxidative stress response of a marine animal. Indeed, given their differing thermal preferences, we suspected that these clonal lines might differ in peroxide tolerance, because thermal stress is known to induce oxidative stress and because ROS such as H_2_O_2_ can work synergistically with acute temperature changes to cause oxidative stress. For example, in the Antarctic limpet *Nacella concina*, H_2_O_2_ exposure and elevated temperatures contribute additively to oxidative stress [47]. Similarly, in corals thermal stress has been shown to result in oxidative DNA damage (e.g., [56]) and to induce antioxidant enzyme activity [51]. Although our study did not directly determine whether there is a correlation between sensitivity to thermal stress and to oxidative stress, the SC clonal line, which was found to be most tolerant of high temperature (29°C; [24]), was among the least affected by H_2_O_2_, and the NS clonal line, which was most sensitive to high temperature, was more affected by H_2_O_2_. However, the Sippewisset clone lines varied extensively in H_2_O_2_ tolerance, and the previous temperature study did not sample enough individuals from this single estuary to determine whether thermal tolerance varies correspondingly within this population.

The most surprising result of our study is the significant differences that we identified in survival and regeneration during H_2_O_2_ exposure among clonal lines taken from a single marsh in Sippewissett, MA. The Sippewissett clonal lines used in our experiments were derived from wild-caught individuals that lived within a hundred meters of each other, some collected from the same small pool where they would have presumably encountered similar environmental stressors; i.e., each of the genets collected from the same pool would have been subjected to the same daily and seasonal variation in temperatures. Given these similar temperature exposures, one would not expect that animals from a single pool would differ widely in their temperature preferences. Nevertheless, the differences among clonal lines from this single pool in their ability to tolerate H_2_O_2_ exposure actually exceeded the variation exhibited between the SC and NS clonal lines. The clonal lines from Sippewisset were collected at the same time and maintained in the laboratory for an equivalent length of time prior to the conduct of this study. Therefore, the differences we observed cannot be explained by differing lengths of time under laboratory culture.

There are multiple possible explanations for the variation we observed within and between pools at Sippewissett. For example, there may be adaptation or long-term acclimation to different microhabitats. Spatially- and temporally-variable factors that can affect peroxide levels include sediment composition and shifting sediment distribution, the amount of organic matter, water depth and tidal action, the amount of pollution and heavy metal contamination, primary production, flooding events (and the associated interconnectedness of tidal pools), aerial exposure, periods of anoxia, solar radiation, and other microbial plant and algal species present [33]. Comparable fine-scale variation in other environmental variables has been shown to affect organismal physiology. For example, substantial environmental micro-variation in temperature has been shown to exist in tide pool habitats [57], and mussels originating only 24 m apart were found to differ substantially in their response to thermal variation [58]. Similarly, changes in vertical gradients of oxygen, H_2_S, temperature, and pH have been shown to effect changes in molecular response to ROS in the burrowing marine polychaete *Heteromastus filiformis* [59]. Gradients of pH have also been shown to affect both oxygen consumption and H_2_O_2_ levels. Previous exposure to sublethal stressors has also been shown to increase stress resistance in a number of model systems an in response to a number of stressors (reviewed in [60]).

Relatively few studies have investigated intraspecific variation in the response to H_2_O_2_, and the bulk of these studies have been performed on plants and algae. For example, intertidal individuals of the green macroalga *Ulva lactuca* exhibited a greater ability to scavenge exogenous H_2_O_2_ than subtidal individuals of the same species [61]. To our knowledge, the only experimental study documenting intraspecific variation in H_2_O_2_ tolerance in an animal model was conducted on the soil nematode *Caenorhabditis remanei* [62]. In this study, multiple strains of *C*. *remanei* were exposed to 3.5 mM H_2_O_2_, an oxidative challenge that kills all individuals within 4-8h of exposure. The offspring of different sires were found to differ in their ability to withstand this level of H_2_O_2_ exposure, and the heritability of this trait was found to be 2x greater than that for heat stress resistance and 11x greater than that for lifespan [62]. This study used a qualitatively different challenge than used in the current study, where the anemones were tested at a level of H_2_O_2_ exposure they could survive for two weeks.

Intraspecific variability in oxidative stress response has been studied in a number of marine invertebrates, including corals and sea anemones. ROS-levels and antioxidant enzyme activities exhibit seasonal variation in the coral *Pocillopora capitata* [63]. In a population of the sea anemone *Anemonia viridis* spanning a natural pCO_2_ gradient, levels of the algal secondary metabolite dimethylsulfoniopropionate (DMSP) as well as the activity of the enzyme superoxide dismutase (SOD) were found to be lower where CO_2_ concentrations were higher [64]. The authors suggest that the symbiotic algae inhabiting the sea anemone’s tissues experience greater photosynthetic efficiency at higher CO_2_ levels, and they therefore generate fewer ROS. In both of these naturalistic studies, the role of ROS could not be isolated from other correlated environmental factors. Nor could these studies discriminate environmental versus genetic causes of the observed phenotypic changes, because it was not known whether the individuals experiencing different environments were part of the same genet or different genets.

Moving forward, an important goal will be to understand the genetic mechanisms (or stably inherited epigenetic mechanisms) that explain the differences in peroxide tolerance observed here. Importantly, according to population genetic studies using AFLPs, the Sippewisset population is genetically distinct from any of the other twenty-three populations that have been studied, including the Baruch, Kingsport, and New Jersey populations [21, 24]. All 36 Sippewisset individuals characterized in these studies appear far more similar to each other than to individuals from any other population [25]. Thus, the most highly resistant clone line from Sippewisset, MA-C2, is much more similar genetically to the least resistant clones line from Sippewissett, MA-A2, than it is to the highly resistant SC clone line. This suggests that differences in oxidative stress tolerance could have evolved independently, and perhaps with a different genetic mechanism, in different populations.

The current study illustrates the importance of incorporating organismal assays into studies of oxidative stress. That an organism is experiencing oxidative stress is often inferred from elevated levels of antioxidant enzymes or non-enzymatic antioxidant defense mechanisms. However, resistant individuals may exhibit greater stress tolerance precisely because they express stress-response genes at higher levels, either constitutively or conditionally (i.e., in response to the relevant stressor) [65]. What ultimately determines the fate of an organism under stress is its ability to maintain physiological function in the face of stressful environmental conditions.

If the degree of fine-scale variability in oxidative stress tolerance observed here in *N*. *vectensis* is widespread among natural populations, it could have profound importance for the resilience of species and ecological communities in this era of rapidly increasing global temperatures because thermal stress is so often coupled with oxidative stress. This is particularly true for reef-building corals. The loss of photosynthetic endosymbiotic zooxanthellae (“bleaching”) is a leading cause of coral mortality. Bleaching is most often associated with elevated temperatures, but the proximate mechanism underlying bleaching is thought to be oxidative stress [66, 67]. As a result, those corals that prove most resistant to thermal bleaching may be those with the most robust anti-oxidant defenses. We suggest that future studies should assess the natural variability in oxidative stress resistance that exists among corals (and other relatively heat-sensitive taxa), and determine if the molecular mechanisms responsible for oxidative stress resistance also confer greater resistance to thermal stress. Such knowledge could prove valuable in developing predictive models for the fate of natural populations in the face of climate change.
